# Intrapatient Variability in Tacrolimus Trough Levels Over 2 Years Affects Long-Term Allograft Outcomes of Kidney Transplantation

**DOI:** 10.3389/fimmu.2021.746013

**Published:** 2021-09-30

**Authors:** Yohan Park, Hanbi Lee, Sang Hun Eum, Hyung Duk Kim, Eun Jeong Ko, Chul Woo Yang, Byung Ha Chung

**Affiliations:** ^1^ Division of Nephrology, Department of Internal Medicine, Konyang University Hospital, College of Medicine, Konyang University, Daejeon, South Korea; ^2^ Transplantation Research Center, Seoul St. Mary’s Hospital, College of Medicine, The Catholic University of Korea, Seoul, South Korea; ^3^ Division of Nephrology, Department of Internal Medicine, Seoul St. Mary’s Hospital, College of Medicine, The Catholic University of Korea, Seoul, South Korea

**Keywords:** allograft, rejection, transplant, graft survival, tacrolimus

## Abstract

This study aimed to determine the impact of tacrolimus (TAC) trough level (C0) intrapatient variability (IPV) over a period of 2 years after kidney transplantation (KT) on allograft outcomes. In total, 1,143 patients with low immunologic risk were enrolled. The time-weighted coefficient variability (TWCV) of TAC-C0 was calculated, and patients were divided into tertile groups (T1: < 24.6%, T2: 24.6%–33.7%, T3: ≥ 33.7%) according to TAC-C0-TWCV up to post-transplant 1^st^ year. They were classified into the low/low, low/high, high/low, and high/high groups based on a TAC-C0-TWCV value of 33.7% during post-transplant 0–1^st^ and 1^st^–2^nd^ years. The allograft outcomes among the three tertile and four TAC-C0-TWCV groups were compared. The T3 group had the highest rate of death-censored allograft loss (DCGL), and T3 was considered an independent risk factor for DCGL. The low/low group had the lowest and the high/high group had the highest risk for DCGL. Moreover, patients with a mean TAC-C0 of ≥5 ng/ml in the high/high group were at the highest risk for DCGL. Thus, TAC-IPV can significantly affect allograft outcomes even with a high mean TAC-C0. Furthermore, to improve allograft outcomes, a low TAC-IPV should be maintained even after the first year of KT.

## Introduction

Tacrolimus (TAC) is the most widely used immunosuppressant drug, and it has better allograft outcomes than other drugs used after kidney transplantation (KT) (1–4). However, it has a narrow therapeutic range. Hence, the monitoring of optimal TAC levels is strongly recommended. At low doses, TAC is associated with high acute rejection rates due to an insufficient immunosuppressive effect (5, 6). Meanwhile, at high doses, it is correlated with adverse events such as infection, malignancy, and nephrotoxicity (7, 8). Therefore, TAC should be administered at an appropriate dose, and patients should undergo therapeutic drug level monitoring (8). Among the indicators for predicting the area under the curve of blood TAC concentration, trough level (C0) has been mainly used for monitoring TAC concentrations in clinical practice (9). Several studies showed that the mean TAC-C0 was significantly associated with clinical outcomes in KT recipients (10, 11).

Meanwhile, variable absorption, first-pass effect, unpredictable metabolism, and, most importantly, nonadherence to TAC can cause fluctuations in TAC-C0 (12, 13). Although the mean TAC-C0 is stable within the target range, there is a risk of extremely low or high drug exposure if there are high fluctuations. In relation to this, previous studies have shown that TAC-C0 intrapatient variability (IPV) can complicate the proper maintenance of the TAC level. Some studies reported that a high TAC-IPV was significantly associated with poor allograft outcomes (14–16). However, most studies have focused on the impact of TAC-IPV during a relatively early post-transplant period, mostly only up to 1 year after KT (14, 16–20).

Only a few studies have reported on TAC-IPV after the first year of KT. In a large-scale study of 6,638 KT recipients, allograft outcomes were poorer if the TAC-IPV was higher according to TAC-C0 at post-transplant first, second, and third years (21). However, because TAC-IPV was calculated using TAC-C0 only at three time points, whether the actual TAC-IPV was represented has been a cause of concern. In another study, TAC-IPV was analyzed at 6-month intervals after KT during a median follow-up period of 3.5 years. The results showed that a high TAC-IPV was correlated with a high risk of allograft loss (22). However, this study used TAC-IPV calculated using the TAC level during the entire study period. Hence, the effect of TAC-IPV during the early *versus* late post-transplant period cannot be differentiated.

Therefore, not only TAC-IPV up to post-transplant first year but also TAC-IPV thereafter may have an important impact on allograft outcomes. However, previous studies about TAC-IPV after the first year of KT had limitations, as mentioned previously. Therefore, the present study aimed to investigate comprehensive allograft outcomes according to TAC-IPV not only up to post-transplant first year but also up to post-transplant second year.

## Materials and Methods

### Study Design

This was a single-center, retrospective observational cohort study that used information collected from a clinical data warehouse system. From January 1996 to December 2018, 1,779 patients received KT at Seoul St. Mary’s Hospital. Patients who experienced allograft loss within 1 year (n = 63), those who died within 1 year (n = 20), those who were lost to follow-up within 1 year (n = 47), those who underwent TAC-C0 measurements <3 times within 1 year (n = 225), those whose treatment was changed from TAC to other drugs within 1 year (n = 166), and those who were sensitized to donor human leukocyte antigen (HLA) before transplantation (n = 115) were excluded. Sensitization to donor HLA was defined as positivity to the complement-dependent cytotoxicity crossmatch test, the flow cytometry crossmatch test, or the presence of donor-specific anti-HLA antibody (HLA-DSA) with a median fluorescence intensity (MFI) of ≥3,000 before transplantation. Finally, 1,143 patients were included in this study. The mean duration of follow-up was 5.7 years.

**Figure 1:**
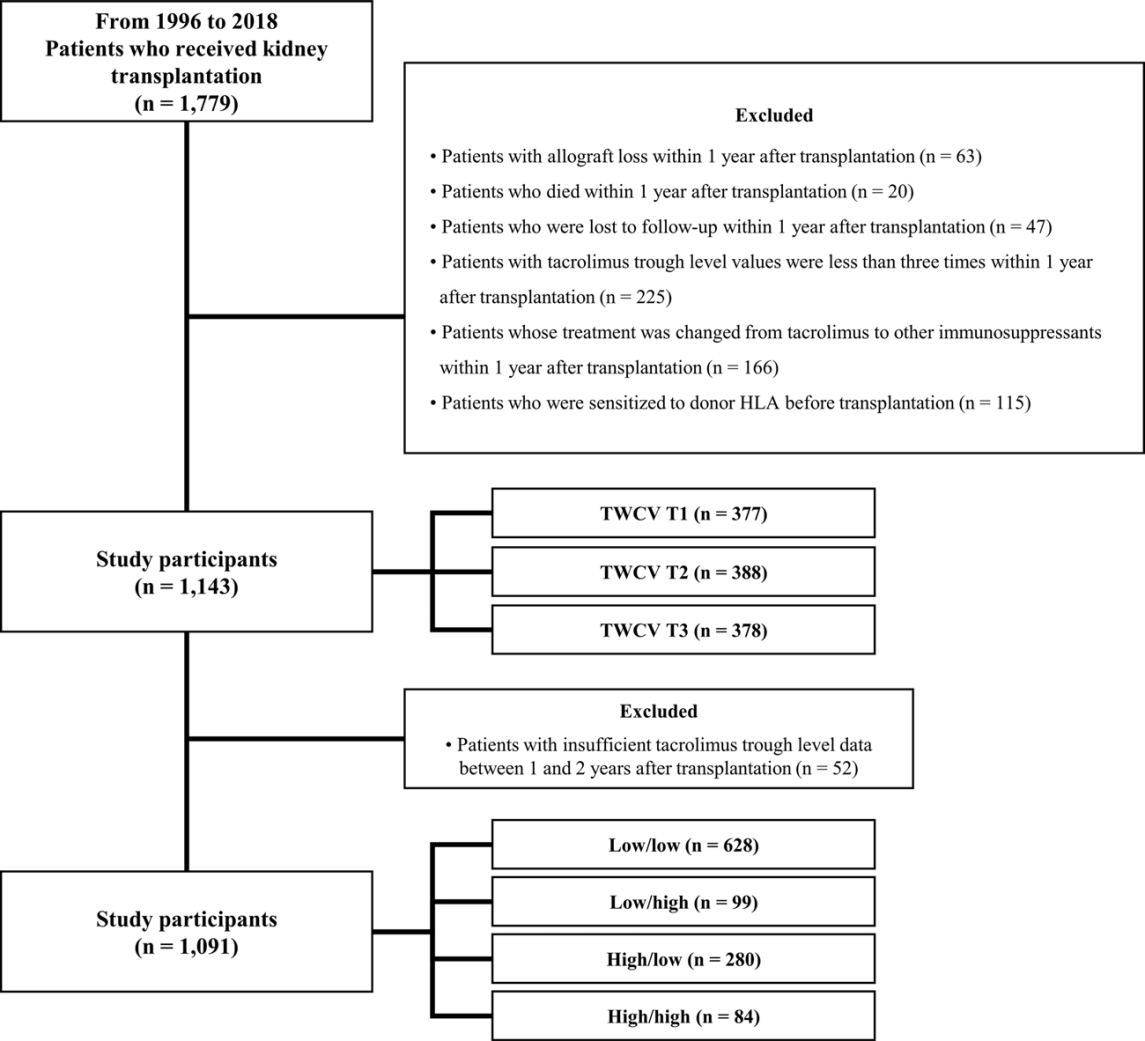
Distribution of patients according to TAC-C0-TWCV. Of 1,779 patients who underwent KT, 636 were excluded. Hence, from January 1996 to December 2018, 1,143 patients were finally included in this study. They were classified into tertile groups according to TAC-C0-TWCV up to post-transplant first year. Fifty-two patients had missing TAC-C0 data during the post-transplant 1^st^–2^nd^ year. In total, 1,091 patients with complete data up to post-transplant second year were classified into four groups according to TAC-C0-TWCV during post-transplant 0–1^st^ and 1^st^–2^nd^ years. HLA, human leukocyte antigen; KT, kidney transplantation; TAC-C0, tacrolimus trough level; TWCV, time-weighted coefficient variability.

TAC-IPV was calculated using the time-weighted coefficient of variability (TWCV), which is described later. The patients were divided into tertile groups (T1, T2, and T3) according to TAC-C0-TWCV up to post-transplant first year. In addition, based on a high TAC-C0-TWCV cutoff value, patients were classified into the low/low, low/high, high/low, and high/high groups according to TAC-C0-TWCV during post-transplant 0–1^st^ and 1^st^–2^nd^ years (**Figure 1**).

This study was performed in accordance with the principles of the Declaration of Helsinki and was approved by the institutional review board of Seoul St. Mary’s Hospital (XC20WIDI0024K).

### TAC-C0-TWCV Calculation

TAC level measurement was performed using the automated Dimension TAC method (Siemens Healthcare Diagnostics Inc, Deerfield, IL), which is an affinity chrome-mediated immunoassay (23). The results of the tests performed in the outpatient department and those conducted just before the next TAC dose in fasting status were used. TAC-C0-TWCV was calculated using a previously reported method (15). Briefly, the time-weighted average (TWA) of TAC-C0 was calculated using the following formula: $\text{TW}µ=\frac{1}{t}\Sigma_{n-1}^{i}x_{i}t_{i}$ . The time-weighted standard deviation was calculated using the following formula: $\text{TW}\sigma =\sqrt{\frac{1}{t}\Sigma_{n=1}^{i}{\left(x_{i}-\mu \right)}^{2}t_{i}}$ , where *i* is the patient’s visit to the *i*th outpatient clinic after transplantation, *x_i_* is the TAC-C0 (ng/ml) during the interval period, *t_i_* is the time interval (days), and *t* is the total duration of drug exposure (days). TAC-C0-TWCV was calculated using the formula $\frac{TW\sigma }{TW\mu }\times 100\;(\%)$ .

### Immunosuppressive Regimen

The maintenance immunosuppressive therapy comprised TAC, mycophenolate mofetil (MMF), and glucocorticoid (prednisolone or deflazacort). The initial TAC was 0.1 mg/kg in two divided doses 2 days before KT. The target TAC-C0 was 8–12 ng/ml until 3 months after KT and 5–8 ng/ml thereafter. The initial MMF was 1,500 mg in two divided doses 2 days before KT. In case of enteric-coated mycophenolate sodium formulation, it was administered at 1,080 mg in two divided doses. Intravenous (IV) glucocorticoid was administered at a high dose during the perioperative period. Then, the dose was reduced (prednisolone 5 mg or deflazacort 6 mg once daily within 3 months after KT). Based on the patient’s immunologic risk (retransplant or positivity to panel reactive antibodies), IV rabbit antithymocyte globulin at a dose of 1.5 mg/kg for 5 consecutive days from day 0 to 4 or anti-interleukin-2 receptor antagonist (basiliximab) at a dose of 20 mg was administered on days 0 and 4. Patients with ABO-incompatible KT received desensitization therapy, as previously reported (24).

### Clinical Parameters

All data were extracted from a clinical data warehouse system. Information about baseline characteristics, including KT donors’ age, sex, and body mass index (BMI); recipients’ age, sex, and BMI; and dialysis- and transplant-related factors, was collected. The concentration-to-dose ratio (CDR) was obtained by dividing TAC-C0 by the previously administered TAC dosage and was used as the average up to post-transplant first year. CDR values were calculated based on the result of the second visit at the outpatient clinic after transplantation. Therefore, the specific period for the CDR values was from a median of 29 days (interquartile range, 27–33 days) to 1 year after transplantation, and the average of these values was obtained and used for analysis. In terms of allograft outcome parameters, data regarding the development of *de novo* DSA, biopsy-proven allograft rejection (BPAR), calcineurin inhibitor (CNI) toxicity, cytomegalovirus (CMV) DNAemia, BK viremia, and death-censored allograft loss (DCGL), as well as mortality rates, were collected.

### Clinical Outcomes

The primary outcome of this study was DCGL, and the secondary outcomes were the development of *de novo* DSA, BPAR, CNI toxicity, and mortality rates.

DCGL was defined as redialysis or retransplantation, excluding patient death with functioning allograft. Mortality was attributed to any cause after transplantation. Allograft kidney biopsy was performed in the case of unexpected allograft dysfunction (serum creatinine level that is 20% above the baseline), unexpected development of proteinuria, and occurrence of *de novo* DSA. Allograft kidney biopsy findings were interpreted according to the 2019 Banff classification. Biopsy-proven rejection was diagnosed *via* allograft biopsy for acute T-cell-mediated rejection (TCMR), acute antibody-mediated rejection (ABMR), chronic active TCMR, and chronic active ABMR. CNI toxicity was diagnosed based on the Banff classification (25, 26). HLA-DSAs were detected using Lifecodes LSA Class I and II kits or LABScreen Single Antigen kit, as previously described (27). A positive result was defined as an MFI of ≥1,000. HLA-DSA monitoring (post-transplant 3–6 and 12 months and annually thereafter) was performed for all patients from January 2010. Moreover, HLA-DSA detection was performed according to the judgment of the clinician when unexpected allograft dysfunction or proteinuria occurred. CMV DNAemia and BK viremia were screened using CMV real-time quantitative (RQ) polymerase chain reaction (PCR) and BK virus real-time (RT) PCR through blood tests at 1- to 2-month intervals up to post-transplant first year. After post-transplant first year, screening was performed with CMV RQ-PCR and BKV RT-PCR every 6 months to 1 year (28, 29). In addition, CMV RQ-PCR and BKV RT-PCR tests were conducted if renal function deteriorated or when the tests were considered necessary per the clinician’s discretion.

### Statistical Analysis

Continuous variables were expressed as mean ± standard deviation. If the variables had a normal distribution, one-way analysis of variance was performed. If the variables had a non-normal distribution, the Kruskal–Wallis test was performed. The independent t-test or Wilcoxon’s rank-sum test followed by the Bonferroni method was performed for *post hoc* analysis. All categorical variables were compared using the chi-square test or Fisher’s exact test and were expressed as proportions. Analysis of death-censored graft survival and patient survival was conducted using Kaplan–Meier curves, and a between-group comparison was performed using the log-rank test. The effect of TAC-C0-TWCV on DCGL was analyzed *via* a Cox proportional hazards regression analysis. We developed a multivariate model with all significant baseline characteristics among the groups. Then, backward selection (likelihood ratio) was applied to eliminate nonsignificant variables (P-value of >0.010). Missing data were censored from the last follow-up date. P-values of <0.05 were considered statistically significant. All statistical analyses were performed using the SAS^®^ version 9.4 software (SAS Institute, Inc., Cary, NC, USA).

## Results

### Comparison of Baseline Characteristics According to TAC-C0-TWCV Tertiles up to Post-transplant First Year

**Table 1** shows the baseline characteristics according to the tertile groups of TWCV calculated using TAC-C0 up to post-transplant first year. In the group classification according to the tertile of TAC-C0-TWCV, the cutoff values of each tertile were 24.6% and 33.7%. The frequency of TAC-C0 measurement was significantly higher in the T3 group than in the other groups. TAC-C0-TWA was highest in the T1 group and lowest in the T3 group. The CDR was highest in the T1 group and lowest in the T3 group (1.99 ± 1.04 in T1, 1.85 ± 1.06 in T2, and 1.77 ± 1.03 in T3, P < 0.001). The T3 group had the lowest proportion of male recipients. The proportion of recipients with positivity to panel reactive antibody (PRA) was highest in the T1 group and lowest in the T3 group.

**Table 1:** Baseline characteristics according to TAC-C0-TWCV tertiles up to post-transplant first year.

	T1	T2	T3	P-value
	(n = 377)	(n = 388)	(n = 378)	
**Donor factors**				
Age (years)	44.2 ± 12.7	44.6 ± 12.6	44.0 ± 12.9	0.821
Male sex	189 (50.1%)	207 (53.4%)	199 (52.7%)	0.647
BMI (kg/m^2^)	23.5 ± 3.4	23.5 ± 3.2	23.6 ± 3.6	0.874
**Recipient factors**				
Tacrolimus measurement times	14.3 ± 2.7^‡^	14.4 ± 2.6^‡^	15.1 ± 2.7^*†^	<0.001
TAC-C0-TWA (ng/ml)	6.76 ± 1.36^†‡^	6.31 ± 1.62*^‡^	6.03 ± 1.80*^†^	<0.001
TAC-C0-TWCV (%)	19.9 ± 3.4^†‡^	28.9 ± 2.6*^‡^	43.3 ± 12.2*^†^	<0.001
CDR	1.99 ± 1.04^‡^	1.85 ± 1.06	1.77 ± 1.03^*^	<0.001
Age (years)	47.2 ± 11.3	46.6 ± 11.3	45.1 ± 11.7	0.046
Male sex	237 (62.9%)	250 (64.4%)	207 (54.8%)	0.014
BMI (kg/m^2^)	23.1 ± 3.6	22.9 ± 3.3	22.9 ± 3.5	0.551
**Cause of ESKD**				
DM	70 (18.6%)	82 (21.1%)	66 (17.5%)	0.413
HTN	59 (15.7%)	52 (10.8%)	61 (16.1%)	0.066
CGN	63 (16.7%)	80 (20.6%)	55 (14.6%)	0.079
Others	116 (30.8%)	123 (31.7%)	117 (31.0%)	0.957
Unknown	69 (18.3%)	61 (15.7%)	79 (20.9%)	0.180
**Dialysis modality**				
Hemodialysis	236 (62.6%)	250 (64.4%)	253 (66.9%)	0.458
Peritoneal dialysis	62 (16.5%)	60 (15.5%)	59 (15.6%)	0.923
Preemptive KT	79 (21.0%)	78 (20.1%)	66 (17.5%)	0.450
Dialysis vintage (months)	52.6 ± 6 2.3	43.6 ± 50.6	53.9 ± 60.2	0.145
**Transplant information**				
Deceased donor KT	129 (34.2%)	131 (33.8%)	149 (39.4%)	0.196
ABO incompatible KT	49 (13.0%)	52 (13.4%)	44 (11.6%)	0.746
Previous KT history	47 (12.5%)	39 (10.1%)	33 (8.7%)	0.234
PRA positive	130 (36.4%)^‡^	104 (28.6%)	85 (25.5%)^*^	0.005
Mismatch number	3.49 ± 1.56	3.55 ± 1.56	3.44 ± 1.50	0.426
**Induction therapy**				
Antithymocyte globulin	63 (16.7%)	67 (17.3%)	48 (12.7%)	0.166
Basiliximab	310 (82.2%)	322 (83.0%)	330 (87.3%)	0.119

Continuous variables are shown as mean ± standard deviation, and categorical variables are shown as proportions. ^*^P < 0.017 versus tertile 1, ^†^P < 0.017 versus tertile 2, ^‡^P < 0.017 versus tertile 3.

BMI, body mass index; CDR, concentration-to-dose ratio; CGN, clinical glomerulonephritis; DM, diabetes mellitus; ESKD, end-stage kidney disease; HTN, hypertension; KT, kidney transplantation; PRA, panel reactive antibody; TAC-C0, tacrolimus trough level; TWA, time-weighted average; TWCV, time-weighted coefficient variability.

### Comparison of the Incidences of BPAR and Other Complications According to TAC-C0-TWCV Tertiles up to Post-transplant First Year

**Table 2** shows the incidence rates of BPAR and other complications according to TAC-C0-TWCV tertiles up to post-transplant first year. The overall BPAR rate was significantly higher in the T3 group. Interestingly, the incidence of acute TCMR significantly differed among the TAC-C0-TWCV tertile groups, whereas that of acute ABMR did not differ among the groups. The incidence of chronic active TCMR did not significantly differ among the groups. However, that of chronic active ABMR significantly differed. The incidence of CNI toxicity was higher with increasing TAC-C0-TWCV tertiles. However, the incidence of *de novo* DSA did not significantly differ among the groups.

**Table 2:** Incidences of BPAR and other complications according to TAC-C0-TWCV tertiles up to post-transplant first year.

	T1	T2	T3	P-value
	(n = 377)	(n = 388)	(n = 378)	
**Overall biopsy-proven rejection**	50 (13.6%)^†^	63 (16.7%)	83 (24.1%)^*^	<0.001
Acute TCMR	39 (10.6%)^†^	49 (13.0%)	69 (20.1%)^*^	0.010
Acute ABMR	9 (2.4%)	11 (2.9%)	11 (3.2%)	0.826
Chronic active TCMR	4 (1.1%)	5 (1.3%)	3 (0.9%)	0.935
Chronic active ABMR	2 (0.5%)^†^	11 (2.9%)	18 (5.2%)^*^	<0.001
***De novo* DSA positive**	43 (12.2%)	45 (12.8%)	30 (11.0%)	0.787
Non-DQ DSA positive	35 (9.9%)	40 (11.3%)	27 (9.9%)	0.772
DQ DSA positive	14 (4.0%)	7 (2.0%)	4 (1.5%)	0.101
**Calcineurin inhibitor toxicity**	50 (13.6%)	68 (18.0%)	70 (20.4%)	0.049
**CMV DNAemia**	63 (16.7%)	71 (18.3%)	79 (20.9%)	0.328
**BK viremia**	50 (13.3%)	73 (18.8%)	56 (14.8%)	0.092

Categorical variables are shown as proportions. ^*^P < 0.017 versus tertile 1, ^†^P < 0.017 versus tertile 3.

ABMR, antibody-mediated rejection; CMV, cytomegalovirus; DSA, donor-specific antibody; TAC-C0, tacrolimus trough level; TCMR, T-cell mediated rejection; TWCV, time-weighted coefficient variability.

### Comparison of the Incidences of BPAR and Other Complications According to TAC-C0-TWCV During Post-transplant 0–1^st^ and 1^st^–2^nd^ Years

**Table 3** shows the incidence rates of BPAR and complications according to TAC-C0-TWCV during post-transplant 0–1^st^ and 1^st^–2^nd^ years. The overall incidence of BPAR was highest in the high/high group and was higher in the low/high and high/low groups than in the low/low group. In a sub-analysis according to rejection type, the incidence of acute ABMR and chronic active TCMR did not significantly differ among the groups. However, the incidence of acute TCMR and chronic active ABMR was significantly higher in the high/high group than in the other groups. The incidence of CNI toxicity was higher in groups with a high TWCV at least once either during post-transplant 0–1^st^ or 1^st^–2^nd^ year (low/high, high/low, and high/high groups *vs.* low/low group).

**Table 3:** Incidences of BPAR and other complications according to TAC-C0-TWCV during post-transplant 0–1^st^ and 1^st^–2^nd^ years.

	Low/low	Low/high	High/low	High/high	P-value
	(n = 628)	(n = 99)	(n = 280)	(n = 84)	
**Overall biopsy-proven rejection**	88 (14.3%)^†^	21 (22.8%)	57 (22.0%)	23 (31.9%)^*^	<0.001
Acute TCMR	70 (11.4%)^†^	15 (16.3%)	45 (17.4%)	21 (29.2%)^*^	<0.001
Acute ABMR	15 (2.4%)	3 (3.3%)	10 (3.9%)	1 (1.4%)	0.592
Chronic active TCMR	7 (1.1%)	2 (2.2%)	1 (0.4%)	2 (2.8%)	0.170
Chronic active ABMR	10 (1.6%)	2 (2.2%)	13 (5.0%)	4 (5.6%)	0.014
***De novo* DSA positive**	68 (11.5%)	12 (16.0%)	26 (12.6%)	3 (5.6%)	0.321
Non-DQ DSA positive	57 (9.6%)	11 (14.7%)	23 (11.1%)	3 (5.6%)	0.341
DQ DSA positive	17 (2.9%)	1 (1.3%)	4 (1.9%)	0 (0%)	0.694
**Calcineurin inhibitor toxicity**	88 (14.3%)	22 (23.9%)	54 (20.9%)	14 (19.4%)	0.026
**CMV DNAemia**	118 (18.8%)	14 (14.1%)	60 (21.4%)	18 (21.4%)	0.414
**BK viremia**	95 (15.1%)	22 (22.2%)	39 (13.9%)	14 (16.7%)	0.254

Categorical variables are shown as proportions. ^*^P < 0.0083 versus low/low group, ^†^P < 0.0083 versus high/high group.

ABMR, antibody-mediated rejection; CMV, cytomegalovirus; DSA, donor-specific antibody; TAC-C0, tacrolimus trough level; TCMR, T-cell mediated rejection; TWCV, time-weighted coefficient variability.

### Comparison of the Incidence of DCGL and Mortality Rates According to TAC-C0-TWCV Tertiles up to Post-transplant First Year

**Figure 2:**
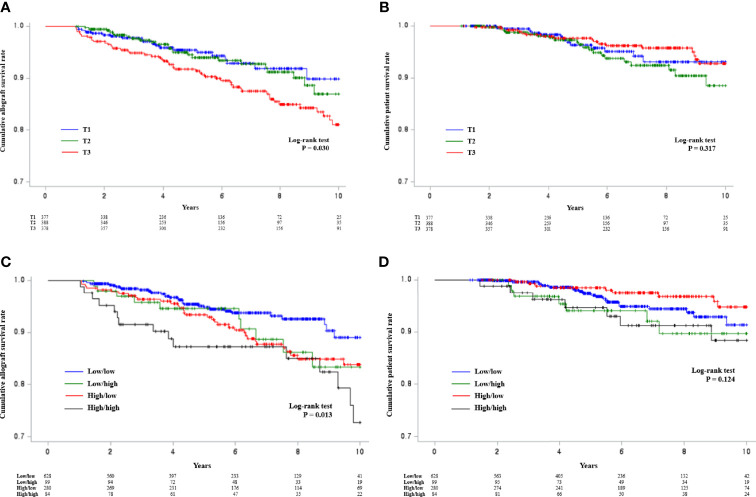
Kaplan–Meier analysis of allograft survival according to TAC-C0-TWCV tertiles up to post-transplant first year and TAC-C0-TWCV tertiles during the post-transplant 0–1^st^ and 1^st^–2^nd^ years. **(A)** The cumulative allograft survival rate was significantly lower in the T3 group than in the other groups. However, there was no difference between the T1 and T2 groups. **(B)** The cumulative patient survival rates did not differ according to the TAC-C0-TWCV tertiles up to post-transplant first year. **(C)** The high/high group had a significantly low cumulative allograft survival rate. In terms of intermediate outcomes, the allograft survival rates of the low/high and high/low groups were similar, and the low/low group had the highest allograft survival rate. **(D)** The cumulative patient survival rates did not differ according to the TAC-C0-TWCV tertiles during the post-transplant 0–1^st^ and 1^st^–2^nd^ years. TAC-C0, tacrolimus trough level; TWCV, time-weighted coefficient variability.

The incidence of DCGL according to TAC-C0-TWCV tertiles up to post-transplant first year was 6.1% (n = 23) in the T1 group, 7.0% (n = 27) in the T2 group, and 14.8% (n = 56) in the T3 group. The incidence of DCGL was significantly higher in the T3 group than in the other groups (P < 0.001, **Supplementary Table S1**). The mortality rates were 3.7% (n = 14) in the T1 group, 5.7% (n = 22) in the T2 group, and 5.0% (n = 19) in the T3 group. However, the results did not significantly differ among the groups (P = 0.437, **Supplementary Table S1**). Using Kaplan–Meier curves, the cumulative allograft survival rate was found to be significantly decreased in the T3 group (**Figure 2A**). The cumulative patient survival rate did not significantly differ among the groups (**Figure 2B**).

### Comparison of the Incidence of DCGL and Mortality Rates According to TAC-C0-TWCV During Post-transplant 0–1^st^ and 1^st^–2^nd^ Years

The incidence rates of DCGL according to TAC-C0-TWCV during post-transplant 0–1^st^ and 1^st^–2^nd^ years were 5.4% (n = 34) in the low/low group, 14.1% (n = 14) in the low/high group, 13.2% (n = 37) in the high/low group, and 20.2% (n = 17) in the high/high group. The high/high group had the highest incidence of DCGL, followed by the low/high or high/low group. Meanwhile, the low/low group had the lowest incidence (P < 0.001, **Supplementary Table S2**). Using Kaplan–Meier curves, the cumulative allograft survival rate was found to be lowest in the high/high group, and the low/high and high/low groups had a similar allograft survival rate, which is an intermediate outcome. The low/low group had the highest allograft survival rate (**Figure 2C**). The mortality rates were 3.5% (n = 22) in the low/low group, 7.1% (n = 7) in the low/high group, 3.2% (n = 9) in the high/low group, and 8.3% (n = 7) in the high/high group. However, the results did not significantly differ among the groups (P = 0.067, **Supplementary Table S2**). Moreover, there was no significant difference in the cumulative patient survival rate among the groups (**Figure 2D**).

### Comparison of Allograft Survival Rates According to TAC-C0-TWA and TAC-C0-TWCV During Post-transplant 0–1^st^ and 1^st^–2^nd^ Years in Each TAC-C0-TWA Subgroup

**Figure 3:**
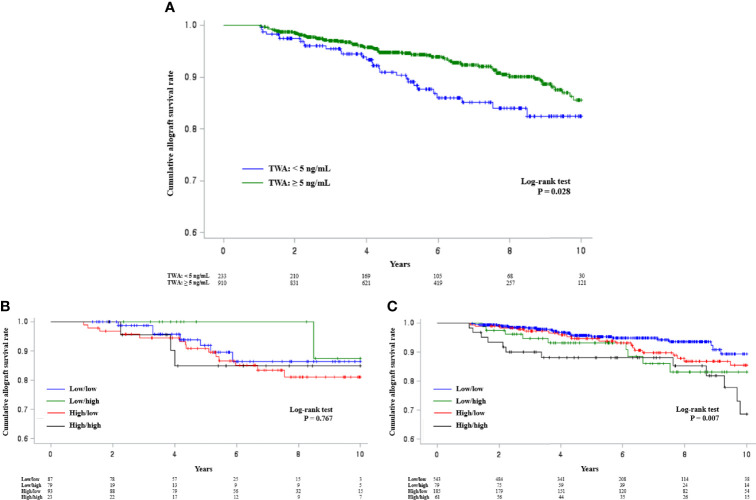
Kaplan–Meier survival analysis of allograft survival according to TAC-C0-TWA and TAC-C0-TWCV during post-transplant 0–1^st^ and 1^st^–2^nd^ years. **(A)** The group with a TAC-C0-TWA of <5 ng/ml had a lower cumulative allograft survival rate than the group with a TAC-C0-TWA of ≥5 ng/ml. **(B)** The cumulative allograft survival rates did not differ among the groups in the subgroup with a TAC-C0-TWA of <5 ng/ml. **(C)** The high/high group in the subgroup with a TWA of ≥5 ng/ml had the lowest cumulative allograft survival rate. These findings were similar to those of the entire patient cohort. TAC-C0, tacrolimus trough level; TWA, time-weighted average; TWCV, time-weighted coefficient variability.

According to a previous report, the risk of allograft loss can increase with a mean TAC-C0 of <5 ng/ml up to post-transplant first year (30). Hence, patients were assessed and then divided into two groups based on a TAC-C0-TWA value of 5 ng/ml up to post-transplant first year. The results showed that the allograft survival rate was significantly poor in the group with a TAC-C0-TWA of <5 ng/ml (**Figure 3A**). The allograft survival rates were analyzed according to TAC-C0-TWCV during post-transplant 0–1^st^ and 1^st^–2^nd^ years in each subgroup. In patients with a TAC-C0-TWA of <5 ng/ml, allograft survival did not differ according to TAC-C0-TWCV levels (**Figure 3B**). However, in patients with a TAC-C0-TWA of ≥5 ng/ml, allograft survival was the worst in the high/high group and the best in the low/low group (**Figure 3C**).

### Multivariate Cox Proportional Hazards Regression Analysis of DCGL

**Table 4** shows the results of multivariate Cox regression analysis of DCGL according to TAC-C0-TWCV up to post-transplant first year (T1, T2, and T3). T3 was an independent risk factor for DCGL, with a hazard ratio (HR) of 1.853 (P = 0.029) after adjusting for the recipient’s age and sex, PRA positivity, CDR, and mismatch number. In the subgroup analysis, in the model for patients with TAC-C0-TWA of ≥5 ng/ml, T3 remained a significant risk factor with HR of 1.932 (P = 0.047) for DCGL. However, in the model for patients with TAC-C0-TWA of <5 ng/ml, it was not observed as a significant risk factor for DCGL. Previous studies have reported that low CDR increases the risk of graft loss (31, 32). There was a significant difference in CDR values among tertile groups at baseline. In this regard, multivariate model analysis including CDR values was performed. In our cohort, CDR was not observed as a significant risk factor for DCGL.

**Table 4:** Multivariate Cox proportional hazard ratio model analysis for DCGL including TAC-C0-TWCV tertiles up to post-transplant first year.

	Univariate HR	P-value		Multivariate HR	P-value
	(95% confidence interval)			(95% confidence interval)	
Entire patient cohort^*^					
**TWCV T1**	Reference	–		Reference	–
**TWCV T2**	1.051 (0.603–1.834)	0.860		1.162 (0.629–2.148)	0.632
**TWCV T3**	1.614 (0.988–2.638)	0.056		1.869 (1.074–3.251)	0.027
**Mismatch number**	1.155 (1.010–1.321)	0.036		1.158 (1.002–1.338)	0.047
**Age**	0.996 (0.978–1.013)	0.627		0.997 (0.978–1.017)	0.768
**Sex (female)**	Reference	–		Reference	–
**Sex (male)**	0.884 (0.602–1.299)	0.532		0.904 (0.585–1.398)	0.651
**PRA positive**	0.636 (0.378–1.070)	0.088		0.647 (0.377–1.110)	0.114
**CDR**	0.906 (0.735–1.116)	0.353		0.955 (0.769–1.186)	0.680
**TAC-C0-TWA ≥ 5 ng/ml** ^†^					
**TWCV T1**	Reference	–		Reference	–
**TWCV T2**	1.132 (0.603–2.126)	0.700		1.357 (0.682–2.700)	0.385
**TWCV T3**	1.630 (0.916–2.900)	0.097		1.932 (1.009–3.701)	0.047
**Mismatch number**	1.141 (0.974–1.338)	0.103		1.161 (0.977–1.379)	0.090
**Age**	0.985 (0.965–1.006)	0.172		0.987 (0.964–1.010)	0.259
**Sex (female)**	Reference	–		Reference	–
**Sex (male)**	0.659 (0.419–1.037)	0.072		0.724 (0.437–1.200)	0.210
**PRA positive**	0.702 (0.386–1.277)	0.247		0.704 (0.378–1.312)	0.270
**CDR**	1.000 (0.800–1.251)	0.997		1.035 (0.820–1.307)	0.772
**TAC-C0-TWA < 5 ng/ml** ^‡^					
**TWCV T1**	Reference	–		Reference	–
**TWCV T2**	0.681 (0.207–2.242)	0.527		0.570 (0.140–2.326)	0.433
**TWCV T3**	0.966 (0.362–2.584)	0.946		0.959 (0.316–2.908)	0.942
**Mismatch number**	1.205 (0.931–1.560)	0.157		1.074 (0.815–1.414)	0.612
**Age**	1.024 (0.990–1.058)	0.164		1.020 (0.982–1.059)	0.300
**Sex (female)**	Reference	–		Reference	–
**Sex (male)**	2.346 (1.099–5.004)	0.027		1.867 (0.741–4.706)	0.186
**PRA positive**	0.497 (0.170–1.449)	0.200		0.632 (0.199–2.008)	0.436
**CDR**	0.680 (0.289–1.600)	0.377		0.830 (0.349–1.971)	0.672

Multivariate model was adjusted with parameters showing significant differences among the groups according to TWCV tertiles during post-transplant first year. ^*^Excluding patients with missing values, 1048 (91.7%) patients were included in the model. ^†^836 (73.1%) patients with TAC-C0-TWA of ≥5 ng/ml were included in the model. ^‡^212 (18.5%) patients with TAC-C0-TWA of <5 ng/ml were included in the model.

CDR, concentration-to-dose ratio; DCGL, death-censored graft loss; PRA, panel reactive antibody; TAC-C0, tacrolimus trough level; TWA, time-weighted average; TWCV, time-weighted coefficient variability.

**Table 5** shows the results of multivariate Cox regression analysis of DCGL according to TAC-C0-TWCV during post-transplant 0–1^st^ and 1^st^–2^nd^ years (low/low, high/low, low/high, and high/high). **Supplementary Table S3** shows the baseline characteristics among the groups, and significant factors were included in the multivariate model (recipient’s age and sex, PRA positivity, basiliximab as an induction therapy, CDR, and mismatch number). The HR of the low/high, high/low, and high/high groups were 2.054 (P = 0.050), 1.818 (P = 0.020), and 2.468 (P = 0.010), respectively, and was the highest in the high/high group. In the subgroup analysis, in the model for patients with TAC-C0-TWA of ≥5 ng/ml, TAC-C0-TWCV during post-transplant 0–1^st^ and 1^st^–2^nd^ years remained a significant risk factor for DCGL [HR of 2.384 (P = 0.027) in the low/high group and HR of 3.084 (P = 0.003) in the high/high group]. However, in the model for patients with TAC-C0-TWA of <5 ng/ml, it was not observed as a significant risk factor for DCGL.

**Table 5:** Multivariate Cox proportional hazard ratio model analysis for DCGL including TAC-C0-TWCV during post-transplant 0–1^st^ and 1^st^–2^nd^ years.

	Univariate HR	P-value	Multivariate HR	P-value
	(95% confidence interval)		(95% confidence interval)	
Entire patient cohort^*^				
**Low/low**	Reference	–	Reference	–
**Low/high**	1.941 (1.038–3.631)	0.038	2.054 (1.000–4.220)	0.050
**High/low**	1.597 (0.997–2.560)	0.052	1.818 (1.098–3.009)	0.020
**High/high**	2.563 (1.424–4.615)	0.002	2.468 (1.243–4.900)	0.010
**Mismatch number**	1.182 (1.030–1.357)	0.017	1.197 (1.032–1.389)	0.018
**Age**	0.996 (0.978–1.014)	0.633	0.995 (0.976–1.016)	0.657
**Sex (female)**	Reference	–	Reference	–
**Sex (male)**	0.909 (0.614–1.346)	0.633	0.937 (0.599–1.467)	0.777
**PRA positive**	0.574 (0.332–0.993)	0.047	0.573 (0.320–1.028)	0.062
**Basiliximab**	1.067 (0.579–1.968)	0.834	0.888 (0.413–1.912)	0.762
**CDR**	0.922 (0.748–1.137)	0.447	0.972 (0.784–1.205)	0.794
**TAC-C0-TWA ≥ 5 ng/ml** ^†^				
**Low/low**	Reference	–	Reference	–
**Low/high**	2.150 (1.064–4.346)	0.033	2.384 (1.103–5.155)	0.027
**High/low**	1.454 (0.819–2.579)	0.201	1.674 (0.904–3.102)	0.102
**High/high**	3.116 (1.622–5.987)	<0.001	3.084 (1.467-6.482)	0.003
**Mismatch number**	1.153 (0.982–1.353)	0.082	1.195 (1.004–1.422)	0.045
**Age**	0.986 (0.966–1.008)	0.207	0.987 (0.674–1.010)	0.270
**Sex (female)**	Reference	–	Reference	–
**Sex (male)**	0.651 (0.412–1.027)	0.065	0.759 (0.454–1.266)	0.291
**PRA positive**	0.705 (0.387–1.283)	0.253	0.681 (0.357–1.298)	0.243
**Basiliximab**	0.915 (0.452–1.853)	0.805	0.777 (0.334–1.808)	0.559
**CDR**	0.991 (0.791–1.242)	0.938	1.008 (0.793–1.281)	0.948
**TAC-C0-TWA < 5 ng/ml** ^‡^				
**Low/low**	Reference	–	Reference	–
**Low/high**	1.152 (0.284–4.676)	0.843	0.676 (0.077–5.930)	0.724
**High/low**	1.310 (0.528–3.249)	0.560	1.312 (0.496–3.469)	0.584
**High/high**	1.010 (0.255–4.008)	0.988	0.514 (0.060–4.393)	0.543
**Mismatch number**	1.279 (0.968–1.689)	0.083	1.107 (0.812–1.509)	0.520
**Age**	1.023 (0.987–1.059)	0.215	1.016 (0.976–1.058)	0.441
**Sex (female)**	Reference	–	Reference	–
**Sex (male)**	2.902 (1.272–6.622)	0.011	2.263 (0.793–6.452)	0.127
**PRA positive**	0.260 (0.061–1.112)	0.069	0.451 (0.093–2.174)	0.321
**Basiliximab**	1.542 (0.444–5.359)	0.495	1.767 (0.228–13.704)	0.586
**CDR**	0.687 (0.279–1.689)	0.413	0.883 (0.360–2.164)	0.786

Multivariate model was adjusted with parameters showing significant differences among the groups according to high or low TWCV during post-transplant 1^st^ and 2^nd^ years. ^*^Excluding patients with missing values, 998 (91.5%) patients were included in the model. ^†^796 (73.0%) patients with TAC-C0-TWA of ≥5 ng/mL were included in the model. ^‡^202 (18.5%) patients with TAC-C0-TWA of <5 ng/mL were included in the model.

CDR, concentration-to-dose ratio; DCGL, death-censored graft loss; PRA, panel reactive antibody; TAC-C0, tacrolimus trough level; TWCV, time-weighted coefficient variability.

## Discussion

This study showed that a high TAC-C0-TWCV not only up to post-transplant first year but also post-transplant first to second year is associated with adverse clinical outcomes such as acute TCMR, chronic ABMR, and DCGL in immunologically low-risk KTs. The results strongly suggest that TAC-IPV should be maintained at least over a period of post-transplant 2 years to improve allograft outcomes.

During the early post-transplant period, the interval of outpatient visits is commonly short. However, over time, the visit interval gradually increases. If the time interval is not kept constant when calculating TAC-IPV, the results might be inaccurate. Therefore, previous studies were generally conducted using TAC-C0 between 6 months and 1 year after KT, when the interval of outpatient clinic visits was constant after KT (14, 17–20). To overcome this limitation, the TAC-C0-TWCV formula was applied to correct for different follow-up intervals. Hence, TAC-C0 during the early period after KT can be used (15). Therefore, in the present study, this formula was employed to analyze parameters, including TAC-C0 in the early period after KT, which can reflect a more accurate TAC-IPV.

Interestingly, the T1 group had the highest TAC-C0-TWA, followed by the T2 group; the T3 group had the lowest TAC-C0-TWA. This may be attributed to the low CDR in the T3 group. A low CDR indicates a fast TAC metabolizer (33). The T3 group had a lower TAC-C0-TWA than the other groups, and this might be attributable to the large proportion of fast TAC metabolizers in this group. In addition, the frequency of TAC-C0 measurement was significantly higher in the T3 group than in the other groups because it may be difficult to reach the therapeutic target range due to large fluctuations in TAC-C0. Hence, more frequent measurements could be performed to assess whether TAC-C0 reached the therapeutic target range. Another possible reason is the high incidences of acute rejection and allograft dysfunction in the T3 group, which could shorten the interval of outpatient clinic visits and resulted in the frequent measurement of TAC-C0 in the T3 group.

Second, we compared the incidence of BPAR and other complications according to TAC-C0-TWCV. The overall BPAR rate was significantly higher in the T3 group than in the other groups. In terms of rejection type, the incidence of acute TCMR significantly differed according to TAC-C0-TWCV tertiles, which was consistent with the results of previous studies (34, 35). Interestingly, the incidence of acute ABMR did not differ, and this might be attributed to the fact that the patients in our study were at low immunological risk. The incidence of chronic ABMR was significantly higher in the T3 group than in the other groups. This result is consistent with previous studies showing that high TAC-IPV was a significant risk factor for composite outcomes, including transplant glomerulopathy and high chronicity scores (16, 19). In addition, the incidence of CNI toxicity was higher in patients with higher TAC-C0-TWCV tertiles, even though the T3 group had a lower TAC-C0-TWA than the T1 group. Therefore, high TAC-IPV is associated with a high risk of not only rejection due to an insufficient immunosuppressive effect but also drug toxicity during exposure to high TAC levels.

In contrast to our expectation, the incidence of *de novo* DSA did not significantly differ according to TAC-C0-TWCV. This could be because not all *de novo* DSA were detected in our study. We previously reported that *de novo* anti-HLA-DQ antibody has the most significant impact on the development of chronic active ABMR (27). However, we could not determine whether the detected anti-HLA-DQ antibody was donor specific before the year 2016 because HLA-DQ typing in kidney donors was started after 2016. The incidence of *de novo* DSA after KT is approximately 15%–25% (36, 37). However, in the present study, the incidence of *de novo* DSA was only 12.0%. Moreover, in the sub-analysis of non-HLA-DQ and HLA-DQ antibodies, the incidence of HLA-DQ antibody was lower than that generally known (38, 39). Thus, the rate of *de novo* anti-HLA-DQ antibody may be significantly underestimated, which might affect the incidence of *de novo* DSA.

The main finding of this study is the impact of TAC-C0-TWCV during post-transplant 0–1^st^ and 1^st^–2^nd^ years on long-term DCGL. Based on an analysis up to post-transplant first year, TAC-C0-TWCV T3 was considered an independent risk factor for DCGL, and the value for defining TAC-C0-TWCV T3 (≥33.7%) was similar to the previously reported TAC-IPV value (≥30%) of poor allograft outcomes (14, 20). Based on this value (≥33.7%), we divided the patients into four groups according to TAC-C0-TWCV during post-transplant 0–1^st^ and 1^st^–2^nd^ years. The patients with a sustained high TAC-C0-TWCV over 2 years after KT (high/high group) had the highest risk for DCGL. Of note, the changes in TAC-C0-TWCV during post-transplant 1^st^–2^nd^ year had a significant influence on allograft outcomes. Indeed, the TAC-C0-TWCV was low up to post-transplant first year, but when it increased during post-transplant 1^st^–2^nd^ year (low/high group), there was a greater risk of DCGL. Conversely, when TAC-C0-TWCV reduced during post-transplant 1^st^–2^nd^ year, even though it was high up to post-transplant first year (high/low group), the risk of DCGL decreased compared with those of the high/high group. Therefore, TAC-IPV even after the first year of KT can significantly influence long-term allograft outcomes.

The Collaborative Transplant Study Registry previously reported that a mean TAC-C0 of <5 ng/ml up to post-transplant first year was associated with a higher incidence of graft loss (30). Expectedly, a TAC-C0-TWA of <5 ng/ml was associated with lower allograft survival in our study. Because TAC-C0-TWA was lowest in the T3 group, whether a lower TAC-C0-TWA worsens allograft outcomes in this group should be considered. However, the TAC-C0-TWA of the T3 group was 6.03 ng/ml, which was higher than the target level of 5 ng/ml. Additionally, in a subgroup analysis of patients with a TAC-C0-TWA of ≥5 ng/ml, allograft survival was the poorest in the high/high group (**Figure 3C**), as shown in the results of the entire patient cohort. In the multivariate analysis of patients with TAC-C0-TWA of ≥5 ng/ml, similar to the results of the entire patient cohort, TAC-C0-TWCV was observed as an independent risk factor for DCGL. In contrast, when only patients with a TAC-C0-TWA of <5 ng/ml were analyzed, there was no significant difference in terms of allograft survival according to TAC-C0-TWCV during post-transplant 0–1^st^ and 1^st^–2^nd^ years (**Figure 3B**). Similarly, in the multivariable analysis of patients with TAC-C0-TWA of <5 ng/ml, TAC-C0-TWCV was not observed as a significant risk factor. The above findings suggest that TAC-C0 should be kept higher than the target level, but even if the mean TAC-C0 was higher than the target level, a high TAC-C0-TWCV can have adverse effects on allograft outcomes. Therefore, not only the TAC-C0 level but also TAC-IPV should be controlled properly.

The present study has several strengths. In a previous study, TAC-C0 was assessed only at three time points (first, second, and third year) after KT (21). Moreover, another research did not utilize TAC-IPV according to a specific period after KT (22). However, the present study calculated TAC-IPV in each period after KT. Notably, the average number of TAC-C0 measurements used to calculate TAC-IPV during the post-transplant 1^st^–2^nd^ year was 7.8 (**Supplementary Table S3**). Hence, the number of measurements was sufficient in reflecting an accurate TAC-IPV. Therefore, our study can provide relatively objective information about the importance of TAC-IPV after the first year of KT.

The present study has several limitations. First, because this study included only immunologically low-risk patients, there is a limit in applying the results of this study to all patients. A previous study reported that TAC-IPV was important even in highly sensitized patients (40). Therefore, we briefly analyzed whether TAC-IPV had an effect even in highly sensitized patients in our cohort. Similar to the previous study, the allograft survival rate tended to be worse in the highest tertile group of TAC-C0-TWCV, but there was no statistical significance owing to the small number of patients (**Supplementary Table S4**, **Supplementary Figure S1**). We are currently conducting further research on this. Second, we did not present the causes for high TAC-C0-TWCV in each patient because the study was retrospective in nature. In this regard, we cannot propose a clear solution for decreasing TAC-C0-TWCV. Third, the directionality between TAC-IPV and treatments remains unclear. Treatments given for rejection might impact TAC-IPV, and the observed higher TAC-IPV might be partially the result of rejection treatment rather than the cause of rejection. However, this is a common limitation of all studies related to TAC-IPV that have been reported to date. High TAC-IPV can be caused by various factors, including drug absorption (41), metabolism (42), formulation (43), concurrent medications (44), and nonadherence among patients (18). Additional studies must be performed to determine whether some interventions, such as patient education, strict monitoring of drug adherence, or use of extended-release TAC formulation, can decrease TAC-IPV and improve allograft outcomes.

In conclusion, TAC-IPV is an important factor that can significantly affect comprehensive allograft outcomes, including rejection, drug toxicity, and allograft loss. In addition, TAC-IPV after the first year of KT was considered an important factor for allograft outcomes. Therefore, continuous control of TAC-IPV, regardless of the post-transplant period, is important in improving allograft outcomes. Further research and clinical efforts to control this IPV are considered to be necessary.

## Data Availability Statement

The raw data supporting the conclusions of this article will be made available by the authors, without undue reservation.

## Ethics Statement

The studies involving human participants were reviewed and approved by Seoul St. Mary’s Hospital (XC20WIDI0024K). Written informed consent for participation was not required for this study in accordance with the national legislation and the institutional requirements.

## Author Contributions

YP and BC wrote the manuscript. YP, CY, and BC designed the study. YP performed the experiments. HL, SE, HK, and EK analyzed the data. All authors contributed to the article and approved the submitted version.

## Funding

This study was supported by a grant from the Korean Health Technology R&D Project, Ministry of Health & Welfare, Republic of Korea (HI20C0317). And this study was supported by Research Fund of Seoul St. Mary's Hospital, the Catholic University of Korea. This study is an investigator led project with no specific participation by the funders.

## Conflict of Interest

The authors declare that the research was conducted in the absence of any commercial or financial relationships that could be construed as a potential conflict of interest.

## Publisher’s Note

All claims expressed in this article are solely those of the authors and do not necessarily represent those of their affiliated organizations, or those of the publisher, the editors and the reviewers. Any product that may be evaluated in this article, or claim that may be made by its manufacturer, is not guaranteed or endorsed by the publisher.
