# Supplementary material for: Intrapatient Variability in Tacrolimus Trough Levels Over 2 Years Affects Long-Term Allograft Outcomes of Kidney Transplantation
Source: Front Immunol. 2021 Sep 30;12:746013. doi: 10.3389/fimmu.2021.746013 (PMC8514869; doi:10.3389/fimmu.2021.746013)
Supplement: Supplementary file 1 [file DataSheet_1.docx]

Supplementary Material

# Supplementary Tables

**Table S1.** DCGL, overall graft loss, and mortality rates according to TAC-C0-TWCV tertiles up to post-transplant 1^st^ year

|  | **T1**  **(n = 377)** | **T2**  **(n = 388)** | **T3**  **(n = 378)** | **P-value** |
| --- | --- | --- | --- | --- |
| **DCGL** | 23 (6.1%)^‡^ | 27 (7.0%)^‡^ | 56 (14.8%)^*†^ | <0.001 |
| **Overall graft loss** | 34 (9.0%)^‡^ | 44 (11.3%) | 68 (18.0%)^*^ | <0.001 |
| **Mortality** | 14 (3.7%) | 22 (5.7%) | 19 (5.0%) | 0.437 |

Categorical variables are shown as proportions. ^*^ P < 0.017 versus tertile 1, ^†^ P < 0.017 versus tertile 2, ^‡^ P < 0.017 versus tertile 3

DCGL, death-censored graft loss; TAC-C0, tacrolimus trough level; TWCV, time-weighted coefficient variability

**Table S2.** DCGL, overall graft loss, and mortality rates according to TAC-C0-TWCV during post-transplant 0–1^st^ and 1^st^–2^nd^ years

|  | **Low/low**  **(n = 628)** | **Low/high**  **(n = 99)** | **High/low**  **(n = 280)** | **High/high**  **(n = 84)** | **P-value** |
| --- | --- | --- | --- | --- | --- |
| **DCGL** | 34 (5.4%)^†‡§^ | 14 (14.1%)^*^ | 37 (13.2%)^*^ | 17 (20.2%)^*^ | <0.001 |
| **Overall graft loss** | 52 (8.3%)^†‡§^ | 20 (20.2%)^*^ | 43 (15.4%)^*^ | 22 (26.2%)^*^ | <0.001 |
| **Mortality** | 22 (3.5%) | 7 (7.1%) | 9 (3.2%) | 7 (8.3%) | 0.067 |

Categorical variables are shown as proportions. ^*^ P < 0.0083 versus low/low group, ^†^ P < 0.0083 versus low/high group, ^‡^ P < 0.0083 versus high/low group, ^§^ P < 0.0083 versus high/high group

DCGL, death-censored graft loss; TAC-C0, tacrolimus trough level; TWCV, time-weighted coefficient variability

**Table S3.** Baseline characteristics according to TAC-C0-TWCV during post-transplant 0–1^st^ and 1^st^–2^nd^ years

|  | **Low/low**  **(n = 628)** | **Low/high**  **(n = 99)** | **High/low**  **(n = 280)** | **High/high**  **(n = 84)** | **P-value** |
| --- | --- | --- | --- | --- | --- |
| **Donor factors** |  |  |  |  |  |
| Age (years) | 44.5 ± 12.6 | 43.4 ± 12.0 | 43.7 ± 13.0 | 44.4 ± 13.0 | 0.769 |
| Male sex | 317 (50.5%) | 56 (56.6%) | 148 (52.9%) | 43 (51.2%) | 0.689 |
| BMI (kg/m^2^) | 23.4 ± 3.2 | 23.8 ± 3.4 | 23.8 ± 3.8 | 23.0 ± 3.3 | 0.406 |
| **Recipient factors** |  |  |  |  |  |
| Tacrolimus measurement times  (0–1 year) | 15.7 ± 2.3 | 14.8 ± 3.4 | 16.1 ± 2.5 | 16.4 ± 2.8 | 0.004 |
| Tacrolimus measurement times  (1–2 years) | 7.5 ± 2.1^†‡§^ | 8.2 ± 2.1^*^ | 8.1 ± 2.5^*^ | 9.0 ± 3.2^*^ | <0.001 |
| TWA (0–1 year) (ng/mL) | 6.54 ± 1.48^‡^ | 6.57 ± 1.86^‡^ | 5.93 ± 1.73^*†^ | 6.28 ± 1.85 | <0.001 |
| TWA (1–2 years) (ng/mL) | 6.23 ± 1.70^‡§^ | 6.52 ± 2.78^‡^ | 4.94 ± 1.87^*†^ | 5.48 ± 2.21^*^ | <0.001 |
| TWCV (0–1 year) (%) | 24.3 ± 5.5^‡§^ | 24.9 ± 5.2^‡§^ | 46.8 ± 11.9^*†^ | 46.4 ± 12.6^*†^ | <0.001 |
| TWCV (1–2 years) (%) | 18.6 ± 6.6^†‡§^ | 46.6 ± 12.9^*‡^ | 21.3 ± 6.4^*†§^ | 46.3 ± 13.3^*‡^ | <0.001 |
| Age (years) | 47.0 ± 11.2 | 47.1 ± 11.9 | 44.7 ± 11.6 | 46.5 ± 11.9 | 0.078 |
| Male sex | 393 (62.6%) | 65 (65.7%) | 160 (57.1%) | 38 (45.2%) | 0.009 |
| BMI (kg/m^2^) | 23.0 ± 3.5 | 22.9 ± 3.2 | 22.9 ± 3.4 | 22.9 ± 3.9 | 0.800 |
| **Cause of ESKD** |  |  |  |  |  |
| DM | 115 (18.3%) | 22 (22.2%) | 45 (16.1%) | 18 (21.4%) | 0.476 |
| HTN | 83 (13.2%) | 13 (13.1%) | 42 (15.0%) | 16 (19.1%) | 0.500 |
| CGN | 120 (19.1%) | 20 (20.2%) | 43 (15.4%) | 11 (13.1%) | 0.316 |
| Others | 195 (31.1%) | 33 (33.3%) | 92 (32.9%) | 24 (28.6%) | 0.853 |
| Unknown | 115 (18.3%) | 11 (11.1%) | 58 (20.7%) | 15 (17.9%) | 0.209 |
| **Dialysis modality** |  |  |  |  |  |
| Hemodialysis | 392 (62.4%) | 65 (65.7%) | 182 (65.0%) | 63 (75.0%) | 0.151 |
| Peritoneal dialysis | 97 (15.5%) | 18 (18.2%) | 42 (15.0%) | 12 (14.3%) | 0.875 |
| Preemptive KT | 139 (22.1%) | 16 (16.2%) | 56 (20.0%) | 9 (10.7%) | 0.067 |
| Dialysis vintage (months) | 47.6 ± 57.5 | 52.9 ± 57.2 | 52.6 ± 59.2 | 57.4 ± 64.4 | 0.282 |
| **Transplant information** |  |  |  |  |  |
| Deceased donor KT | 204 (32.5%) | 41 (41.4%) | 104 (37.1%) | 37 (44.1%) | 0.071 |
| ABO-incompatible KT | 89 (14.2%) | 11 (11.1%) | 30 (10.7%) | 14 (16.7%) | 0.350 |
| Previous history of KT | 73 (11.6%) | 12 (12.1%) | 26 (9.3%) | 6 (7.1%) | 0.482 |
| PRA positive | 194 (32.5%) | 30 (34.5%) | 64 (25.6%) | 18 (25.4%) | 0.139 |
| Mismatch number | 3.56 ± 1.55 | 3.29 ± 1.61 | 3.46 ± 1.56 | 3.37 ± 1.37 | 0.274 |
| **Induction therapy** |  |  |  |  |  |
| Antithymocyte globulin | 109 (17.4%) | 17 (17.2%) | 33 (11.8%) | 11 (13.1%) | 0.161 |
| Basiliximab | 521 (83.0%) | 77 (77.8%) | 249 (88.9%) | 72 (85.7%) | 0.034 |

Continuous variables are shown as mean ± standard deviation and categorical variables are shown as proportions. ^*^ P < 0.0083 versus low/low group, ^†^ P < 0.0083 versus low/high group, ^‡^ P < 0.0083 versus high/low group, ^§^ P < 0.0083 versus high/high group

BMI, body mass index; CGN, clinical glomerulonephritis; DM, diabetes mellitus; ESKD, end-stage kidney disease; HTN, hypertension; KT, kidney transplantation; PRA, panel reactive antibody; TAC-C0, tacrolimus trough level; TWA, time-weighted average; TWCV, time-weighted coefficient variability

**Table S4.** DCGL, overall graft loss, and mortality rates according to TAC-C0-TWCV tertiles up to post-transplant 1^st^ year in highly sensitized patients

|  | **T1^*^**  **(n = 42)** | **T2^*^**  **(n = 39)** | **T3^*^**  **(n = 34)** | **P-value** |
| --- | --- | --- | --- | --- |
| **DCGL** | 1 (2.4%) | 2 (5.1%) | 6 (17.7%) | 0.061 |
| **Overall graft loss** | 2 (4.8%) | 3 (7.7%) | 9 (26.5%) | 0.014 |
| **Mortality** | 2 (4.8%) | 2 (5.1%) | 5 (14.7%) | 0.254 |

Categorical variables are shown as proportions. ^*^ The cutoff values of TAC-C0-TWCV of each tertile were 24.0% and 35.5%.

DCGL, death-censored graft loss; TAC-C0, tacrolimus trough level; TWCV, time-weighted coefficient variability

# Supplementary Figure legends

**Figure S1. Kaplan–Meier analysis of allograft survival according to TAC-C0-TWCV tertiles up to post-transplant 1^st^ year in highly sensitized patients**

The cumulative allograft survival rate tended to be worse in the T3 group but there was no statistical significance.
