# Supplementary figures and images for: Intrapatient Variability in Tacrolimus Trough Levels Over 2 Years Affects Long-Term Allograft Outcomes of Kidney Transplantation
Source: Front Immunol. 2021 Sep 30;12:746013. doi: 10.3389/fimmu.2021.746013 (PMC8514869; doi:10.3389/fimmu.2021.746013)

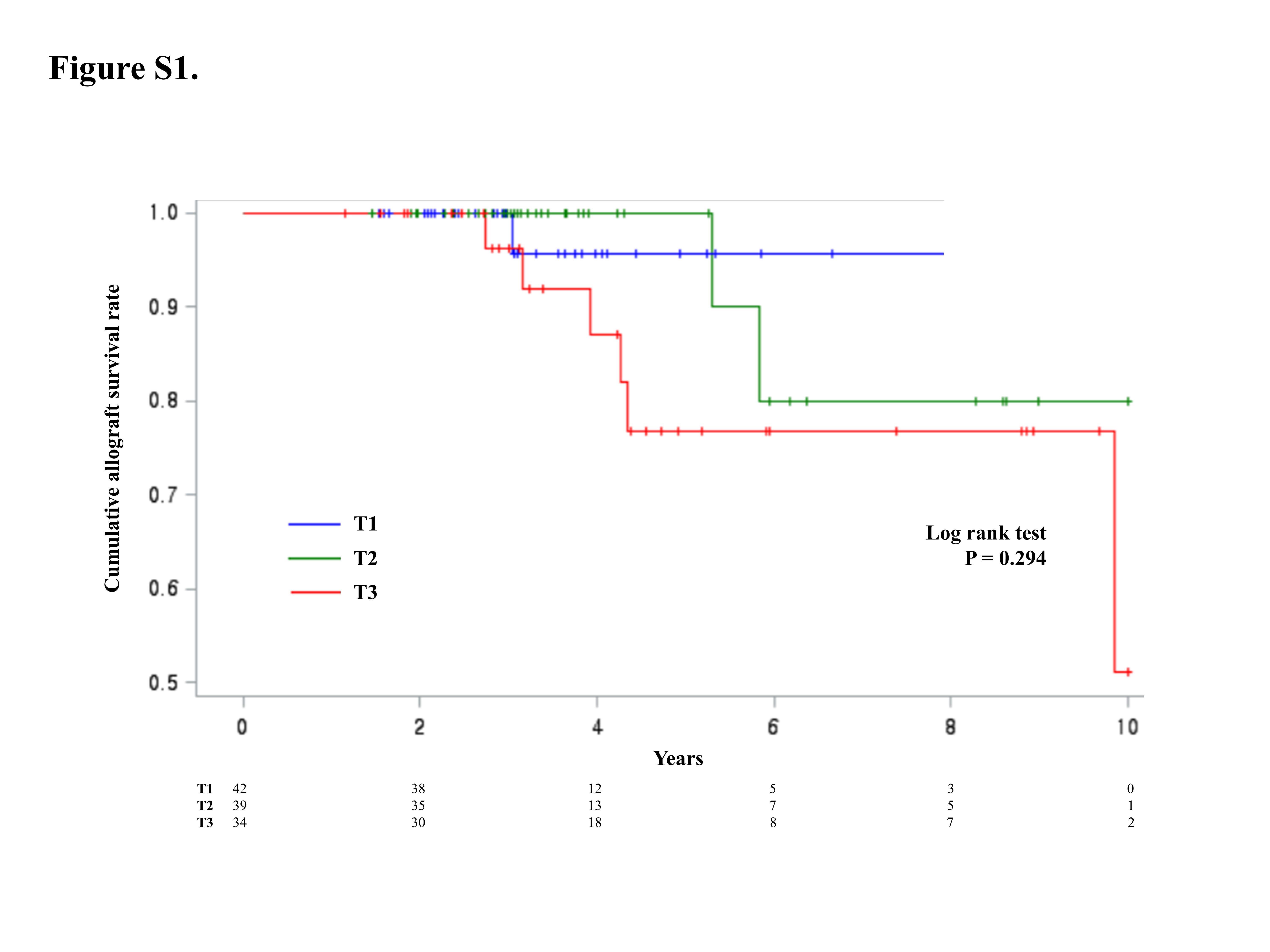

Supplement: Supplementary file 2 [file Image_1.tif]
